# The Association of Sleep Disorder, Obesity Status, and Diabetes Mellitus among US Adults—The NHANES 2009-2010 Survey Results

**DOI:** 10.1155/2013/234129

**Published:** 2013-07-16

**Authors:** Jian Liu, John Hay, Brent E. Faught

**Affiliations:** Brock University, 500 Glenridge Avenue, St. Catharines, ON, Canada L2T 3A1

## Abstract

To examine the association between sleep disorders, obesity status, and the risk of diabetes in adults, a total of 3668 individuals aged 40+ years from the NHANES 2009-2010 without missing information on sleep-related questions, measurements related to diabetes, and BMI were included in this analysis. Subjects were categorized into three sleep groups based on two sleep questions: (a) no sleep problems; (b) sleep disturbance; and (c) sleep disorder. Diabetes was defined as having one of a diagnosis from a physician; an overnight fasting glucose > 125 mg/dL; Glycohemoglobin > 6.4%; or an oral glucose tolerance test > 199 mg/dL. Overall, 19% of subjects were diabetics, 37% were obese, and 32% had either sleep disturbance or sleep disorder. Using multiple logistic regression models adjusting for covariates without including BMI, the odds ratios (OR, (95% CI)) of diabetes were 1.40 (1.06, 1.84) and 2.04 (1.40, 2.95) for those with sleep disturbance and with sleep disorder, respectively. When further adjusting for BMI, the ORs were similar for those with sleep disturbance 1.36 (1.06, 1.73) but greatly attenuated for those with sleep disorders (1.38 [0.95, 2.00]). In conclusion, the impact of sleep disorders on diabetes may be explained through the individuals' obesity status.

## 1. Introduction

Over the past two decades, a dramatic increase in prevalence of obesity has been witnessed globally. As the result, the incidence and prevalence rates of type 2 diabetes (T2D) have also been elevated significantly. In the United States, it has been estimated that currently approximately a third of adults are obese (defined as BMI ≥ 30 kg/m^2^) [1], and over 11% of people aged 20 years and older have diabetes [2]. Among those who are obese, approximately 60% have T2D [3]. The increasingly common early onset of T2D is not surprising as more children become overweight and obese [4]. The precise mechanisms linking obesity status and T2D remain unclear, but the progress of insulin resistance is considered as the key to this link [5]. Sleep problems are common among those obese subjects who are at a great risk for developing T2D [6]. The emerging evidence indicates that sleep-related problems may play a role in the development of these concurrent illnesses [7]. A clear understanding of the interrelationships between sleep-related problems, obesity, and T2D may provide information that would allow effective strategies for prevention and/or intervention of these two diseases. In this report, the National Health and Nutrition Examination Survey (NHANES) 2009-2010 data was used to examine the associations between sleep status, obesity status, and T2D.

## 2. Methods

### 2.1. NHANES 2009-2010 and Its Participants

The NHANES 2009-2010 is a stratified, multistage probability design survey conducted between 2009 and 2010 with approximately 10,500 people aged 0 year and over being involved. Details of the survey design and measurement procedures can be found elsewhere [8]. We restricted our sample to those whose age was 40 years and older since T2D is the most common type of diabetes mellitus among adults and diabetes among individuals aged 40 and above is most likely T2D. A total of 4,009 subjects aged 40 years and older were identified in the NHANES 2009-2010 data set. After removing individuals with missing information on sleep-related questions, variables related to diabetes, body mass index, and pregnant women, a total of 3,668 individuals (1,813 men and 1,855 women) were included in this analysis. 

### 2.2. Sleep Disorders Measurements

Three sleep-related questions were asked, in the home, by trained interviewers using the Computer-Assisted Personal Interviewing (CAPI) system. They are “how much sleep do you usually get at night on weekdays or workdays?” “Have you ever told a doctor or other health professionals that you have trouble sleeping?” and “Have you ever been told by a doctor or other health professional that you have a sleep disorder?” The first question was measured by hours in sleep and the other two questions as “yes” versus “no” responses. Using the last two questions, all subjects were grouped into three categories; that is, group one, no sleep problems if the answers for both questions were “no”; group two, having a sleep disturbance if “yes” to the first question, but “no” to the second question; and group three, having a sleep disorder if “yes” to the second question. Since it has been suggested that either shorter sleep hours or longer sleep hours are associated with the increased risk for cardiovascular disease [9], sleeping hour responses are also categorized into three groups <7, 7-8, and >8 hours. 

### 2.3. Body Mass Index Related Measurements

Body mass index (BMI) was derived from body weight and standing height and was used to determine obesity status of the participants in the survey. Both weight and height are part of body measurements, which were measured in the Mobile Examination Center (MEC), by trained health technicians following the 2009-2010 NHANES anthropometry protocol [10]. Participants wore the standard MEC examination gown, which consists of a disposable shirt, pants, and slippers. Weight was measured using a digital weight scale and measured to near 0.1 kg. Standing height was measured using a stadiometer and measured to 0.1 cm. BMI was calculated as weight (kg) divided by the square of their standing height (m^2^) and then categorized into three groups, that is, BMI < 25.0 kg/m^2^, BMI: 25.0–29.9 kg/m^2^, and BMI ≥ 30.0 kg/m^2^. The last two groups were considered as overweight and obese, respectively [11]. There are 95 subjects (51 men and 44 women), about 2.6% of the study sample, whose BMI were below 18.5 kg/m^2^, which is considered as underweight [12]. Similar results are found when excluding these subjects; therefore, we reported our results with them included.

### 2.4. Diabetes Mellitus Related Measurements

Diabetes mellitus is defined as either having been diagnosed with diabetes mellitus by a physician, or a fasting glucose level >125 mg/dL (6.9 mmol/L), or a 2-hour value in the oral glucose tolerance test (OGTT) >199 mg/dL (11.1 mmol/L), or HbA1C level ≥6.5% [13]. Blood was drawn at the MEC, and blood specimens were processed, stored, and shipped to Fairview Medical Center Laboratory at the University of Minnesota, Minneapolis Minnesota for analysis. The concentration of glucose was measured using enzymatic method, and the level of HbA1C was measured using G7 Glycohemoglobin Analyzer [14]. Diabetes mellitus was the main outcome and was coded as 1 = yes and 0 = no in the analysis.

### 2.5. Measurements of Covariates

Covariates in the analysis included age (years), gender (male versus female), ethnicity (non-Hispanic white versus others), education (less than high-school versus high-school or higher), marital status (living with a spouse or partner: yes versus no), ratio of family income to poverty threshold, currently cigarette smoking (yes versus no), alcohol drinking (average drinks per day), sedentary activity time per day (minutes), total to HDL cholesterol ratio, systolic blood pressure (mmHg), and C-reactive protein (mg/dL). 

### 2.6. Statistical Analysis

All analyses were conducted using survey procedures in SAS 9.3 (SAS Institute Inc., Cary, NC, USA), and take into account the weighted and clustered sampling design of NHANES. The significant level was defined at 2-tailed alpha equal or less than 0.05. Odds ratio (OR) and its 95% confidence interval (CI) from logistic regression models were used to examine the relationship between diabetes and sleep disorder status, and four models have been used for this purpose. Diabetes mellitus is the dependent variable in each model, and two indicator variables were created for people having a sleep disturbance and having a sleep disorder, respectively. Subjects who reported no sleep issues are considered as the reference in the analyses. The covariates in Model One were age, gender, race, education, ratio of family income to poverty, and marital status; in Model Two were the covariates in Model One plus total to HDL cholesterol ratio, systolic blood pressure, sedentary active time, alcohol drinking, and cigarette smoking; in Model Three were the covariates in Model Two plus C-reactive protein and sleep duration; and in Model Four, BMI was added to the analysis. 

 To examine the impact of interaction of sleep disorder and obesity status on diabetes mellitus, we further created eight indictors to indicate people's sleep disorder and obesity status, that is, (1) reporting a sleep disturbance and BMI < 25.0 kg/m^2^; (2) reporting a sleep disorder and BMI < 25.0 kg/m^2^; (3) reporting no sleep problems and BMI: 25.0–29.9 kg/m^2^; (4) reporting a sleep disturbance and BMI: 25.0–29.9 kg/m^2^; (5) reporting a sleep disorder and BMI: 25.0–29.9 kg/m^2^; (6) reporting no sleep problem and BMI ≥ 30.0 kg/m^2^; (7) reporting a sleep disturbance and BMI ≥ 30 kg/m^2^; and (8) reporting a sleep disorder and BMI ≥ 30 kg/m^2^. Those people reporting no sleep issues and BMI < 25 kg/m^2^ were used as the reference, and all covariates in the Model Three in previous analyses were included. 

## 3. Results

Approximately 19% individuals in this sample were categorized as diabetes mellitus. The prevalence rates of having sleep disturbance and sleep disorder were 22.1% and 9.4%, respectively. Based on BMI measurements, approximately 35% of people were in the overweight group and 37% were in obese group. Characteristics of participants by sleep disorder status are shown in Table 1. Compared to people without sleep problem, individuals with sleep disturbance had similar profiles apart from being more likely to be female, non-Hispanic white, less likely to live with a spouse or partner, having a higher level of C-reactive protein, and shorter sleep duration. Those having sleep disorder were statistically different from those without sleep problems except for age, ratio of family income to poverty, lifestyle variables, total to HDL cholesterol ratio, blood pressure measurements, and percentage male.

The ORs and their 95% CIs derived from logistic regression models are presented in Table 2. The odds of diabetes for the first three models are similar. Compared to those having no sleep problem, the ORs of diabetes in Model Three for those with a sleep disturbance and those with a sleep disorder were 1.40 (95% CI: 1.06, 1.84) and 2.04 (95% CI: 1.40, 2.95), respectively. When further adjusting for BMI in Model Four, the OR for those with a sleep disturbance did not change much (OR (95% CI): 1.36 (1.06, 1.73)) but was greatly attenuated for those having a sleep disorder (1.38 (0.95, 2.00)). 

**Figure 1:**
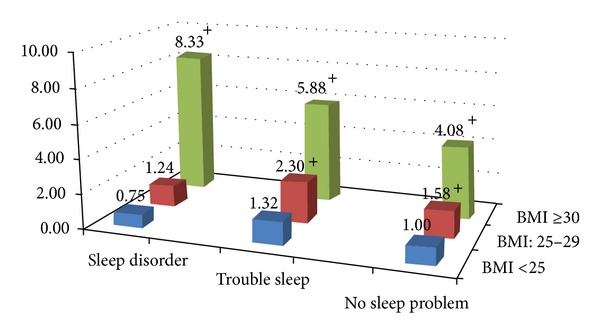
The ORs of diabetes by different combinations of sleep and obesity status after adjustment for age, gender, race, education, ratio of family income to poverty, marital status, total to HDL cholesterol ratio, systolic blood pressure, sedentary activity time, alcohol drinking, cigarette smoking, C-reactive protein, and sleep duration. The group of BMI < 25.0 with no sleep problem is the reference group. ^+^ *P* value <0.05.

Figure 1 shows the ORs of diabetes for different combinations of sleep and obesity status. After adjustment for all covariates used in Model three in the previous analysis and using those reporting no sleep problems and BMI < 25 kg/m^2^ as the referent group, the odds of diabetes for those with a sleep disturbance or a sleep disorder were similar if their BMI was <25 kg/m^2^. The ORs (95% CIs) of T2D for sleep disturbance and sleep disorder were 1.32(0.69, 2.49) and 0.75 (0.28, 1.99), respectively, when they were in the group of BMI < 25 kg/m^2^. The ORs (95% CIs) for those with BMI: 25.0–29.9 kg/m^2^ were 1.58 (1.16, 2.15) in the group of reporting no sleep problem, 2.30 (1.37, 3.86) in the group of reporting a sleep disturbance, and 1.24 (0.57, 2.68) in the group of reporting a sleep disorder. While the ORs (95% CIs) for those with a BMI ≥ 30 kg/m^2^ were 4.08 (2.59, 6.41), 5.88 (3.53, 9.80), and 8.33 (4.81, 14.49) for those in the groups of reporting no sleep problem, a sleep disturbance, and a sleep disorder, respectively.

 To confirm that the results from the previous analyses were not due to the change of the sample size from model to model, we used a multiple imputation method [15] to create a database with all missing values imputed, and the results are similar (data not shown).

## 4. Discussion

Although evidence indicates that sleep problems increase the risk of cardiovascular disease (CVD) [16–18], and a similar risk association has been observed between sleep problems and diabetes mellitus [19–21], it is unclear whether the impact of sleep problems on T2D may be mediated through obesity. Because sleep disorders are prevalent among people with overweight and obesity, which is also a risk for T2D [22–24]; when examining the risk association of T2D with sleep problems in cohort studies, researchers previously took into account the impact of BMI measured at baseline [25–28]. Yet, the results from these cohort studies are not consistent. For instance, a cohort of women from Gothenburg, Sweden, were followed for 32 years, and no significant associations or trends were observed between incidence of diabetes and any of the available sleep indicators; however, obesity status, using either BMI or waist circumference, was highly associated with sleep problems [26]. The results from the Nurses Health Study suggested that sleep restriction might mediate the effect of weight change on diabetes mellitus [25]. However to date, no study has closely examined these associations. Using recent NHANES data, we explored this issue and found that people with sleep disorders are indeed associated with a great risk of diabetes mellitus independently from a number of known risk factors without including BMI. However, after taking BMI into account the odds ratio of diabetes mellitus is similar for those with sleep disturbance but significantly attenuated for those with sleep disorders. This suggests that the impact of sleep disorders on diabetes mellitus may be confounded by BMI. Results from the model with different combinations of sleep and obesity status (Figure 1) further demonstrate that the risk association of diabetes mellitus with sleep disorders may be mediated through obese status. Subjects not in the overweight or obese groups but having a sleep disturbance or disorder had similar odds for diabetes mellitus as those with no sleep problems. However, for those in the overweight group (BMI: 25–29.9 kg/m^2^), the odds of T2D are higher regardless of their sleep status, while those in the obese group had over four folds higher odds of T2D and those with a sleep disorder had the highest one. 

 Laboratory studies conducted among healthy adults have shown that short-term sleep deprivation could increase each of insulin resistance, sympathetic tone, blood pressure, and C-reactive protein level [29–31]. Observational studies have reported that children with sleep problems are more likely to be overweight and obese [32, 33]. It appears that having a healthy weight through exercise may be the key variable in both overweight/obesity prevention and intervention [34] and that the preschool years are a key time for shaping relevant attitudes and behaviours [35]. As physical activity also has a positive effect on sleep pattern, its benefits may go beyond simple weight control [36, 37]. Although in this report, we cannot test the role of physical activity on the association between sleep disorders, obesity status, and diabetes mellitus, the significantly longer sedentary time among those individuals with sleep disorders in comparison to those reporting no sleep problems suggests that physical activity is a critical element for intervention. 

 Compared to waist circumference, BMI has been criticized for not being a good indicator of central obesity in the risk prediction of cardiovascular disease [38]. Central obesity is a prevalent trait among people with sleep disorders, but results from a meta-analysis indicated that both BMI and waist circumference are similarly associated with the risk of diabetes since they are highly correlated to each other [39]. The Pearson correlation coefficient between the body mass index and waist circumference in our data is 0.9, and the replacement of BMI with waist circumference in the Model 4 shows similar results—ORs (95% CI) of diabetes for sleep disturbance and sleep disorders are 1.31 (1.01, 1.68) and 1.28 (0.88, 1.85), respectively. This suggests that the impact of sleep problems on diabetes is indeed affected by individual's obesity status though different sleep problems show different interactions.

 Several limitations must be recognized when interpreting the results of this study. First, this was a cross-sectional study, and therefore conclusions about causal associations cannot be made. Second, the sleep-related problems were self-reported and may not reflect the true picture of sleep status. However, the results from a study indicate that self-reported sleep duration was relatively accurate [40]. The “yes/no” questions on whether one has reported having trouble sleeping to a physician or whether a physician has told the person that he or she has a sleep disorder are quite straightforward and should be reliable. Third, we did not control for the impact of physical activity in this study, but we did control for the self-reported sedentary activity time, which may be underreported due to a social desirability bias. However, we assume that this underreported time is random and should not differ with different sleep status. The strengths of this study are (1) the data are from a well-designed nationally representative survey; (2) the laboratory measurements are validated and reliable; and (3) we have adjusted for a number of known risk factors including sleeping duration. 

 In conclusion, sleep disturbance is independently associated with an increased risk for T2D. However, the impact of sleep disorders on T2D may be explained by obesity status. Longitudinal research is needed to examine the relationship between sleep problems, changes in obesity status, and their impacts on the development of T2D.

## Figures and Tables

**Table 1:** Basic characteristics of 3668 participants in the NHANES 2009-2010 by sleep disorder status.

	None	Sleep disturbance	Sleep disorders
*n*	2551	773	344
Demographic measures			
Age (yrs, mean [S.E.])	57.9 [0.3]	58.2 [0.7]	56.9 [0.7]
Male (%)	50.5	37.3^∧^	51.5
Non-Hispanic white (%)	72.1	79.9^∧^	76.3^∧^
Less than high school (%)	20.9	19.6	17.6^∧^
Living with spouse or partner (%)	70.9	63.2^∧^	61.3^∧^
Ratio of family income to poverty (mean [S.E.])	3.23 [0.04]	3.17 [0.09]	3.1 [0.14]
Cigarette smoking (%)	14.4	15.6	14.9
Alcohol drinking (average drinks/day, mean (S.E.])	1.9 [0.4]	1.4 [0.1]	1.5 [0.14]
Body composition measures			
Body mass index (kg/m^2^, mean [S.E.])	28.5 [0.2]	29.3 [0.3]	32.9 [0.5]^∧^
Waist circumference (cm, mean [S.E.])	99.2 [0.5]	100.8 [0.8]	109.4 [1.3]^∧^
Measurements from blood sample			
Total to HDL cholesterol ratio (mean [S.E.])	4.06 [0.03]	4.0 [0.06]	4.1 [0.11]
C-reactive protein (mg/dL, mean [S.E.])	0.33 [0.01]	0.51 [0.06]^∧^	0.54 [0.06]^∧^
Glycohemoglobin (%, mean [S.E.])	5.77 [0.03]	5.86 [0.04]	5.98 [0.06]^∧^
Blood pressure and pulse measures			
Systolic blood pressure (mmHg, mean [S.E.])	124.7 [0.6]	125.1 [0.9]	125.0 [1.3]
Diastolic blood pressure (mmHg, mean [S.E.])	70.1 [0.7]	69.8 [0.7]	69.8 [1.3]
Sedentary activity (min, mean [S.E.])	336.4 [6.0]	351.9 [10.7]	389.9 [11.6]^∧^
Sleep duration (hours, mean [S.E.])	7.09 [0.03]	6.51 [0.08]^∧^	6.54 [0.11]^∧^
Diabetes (%)	17.1	20.9	27.2^∧^

^∧^ *P* < 0.05 when compared to “none.”

**Table 2:** Adjusted odds ratio of diabetes for sleep disorder status, NHANES 2009-2010.

	*N*	None	OR (95% confidence interval)	
			With a sleep disturbance	With a sleep disorder
Model 1	3300	1.00	1.43 (1.07, 1.89)	2.08 (1.39, 3.09)
Model 2	3188	1.00	1.44 (1.05, 1.99)	2.14 (1.44, 3.17)
Model 3	3185	1.00	1.40 (1.06, 1.84)	2.04 (1.40, 2.95)
Model 4	3148	1.00	1.36 (1.06, 1.73)	1.38 (0.95, 2.00)

Model 1: adjustment for age, gender, race, education, ratio of family income to poverty, and marital status.

Model 2: further adjustment for total to HDL cholesterol ratio, systolic blood pressure, sedentary activity time, alcohol drinking, and cigarette smoking.

Model 3: further adjustment for C-reactive protein and sleep duration.

Model 4: further adjustment for BMI.
